# A highly effective ferritin-based divalent nanoparticle vaccine shields Syrian hamsters against lethal Nipah virus

**DOI:** 10.3389/fimmu.2024.1387811

**Published:** 2024-06-06

**Authors:** Chunhong Yin, Yan Feng Yao, Peipei Yang, Hang Liu, Ge Gao, Yun Peng, Miaoyu Chen, Mingqing Lu, Xuekai Zhang, Weiwei Guo, Zihan Zhang, Xue Hu, Zhiming Yuan, Chao Shan

**Affiliations:** ^1^ State Key Laboratory of Virology, Wuhan Institute of Virology, Chinese Academy of Sciences, Wuhan, China; ^2^ Center for Biosafety Mega-Science, Wuhan Institute of Virology, Chinese Academy of Sciences, Wuhan, China; ^3^ University of the Chinese Academy of Sciences, Beijing, China; ^4^ Hubei Jiangxia Laboratory, Wuhan, China

**Keywords:** Nipah virus, nanoparticle vaccine, attachment glycoproteins (G), divalent vaccine, immune responses, cross-reactivity

## Abstract

The Nipah virus (NiV), a highly deadly bat-borne paramyxovirus, poses a substantial threat due to recurrent outbreaks in specific regions, causing severe respiratory and neurological diseases with high morbidity. Two distinct strains, NiV-Malaysia (NiV-M) and NiV-Bangladesh (NiV-B), contribute to outbreaks in different geographical areas. Currently, there are no commercially licensed vaccines or drugs available for prevention or treatment. In response to this urgent need for protection against NiV and related *henipaviruses* infections, we developed a novel homotypic virus-like nanoparticle (VLP) vaccine co-displaying NiV attachment glycoproteins (G) from both strains, utilizing the self-assembling properties of ferritin protein. In comparison to the NiV G subunit vaccine, our nanoparticle vaccine elicited significantly higher levels of neutralizing antibodies and provided complete protection against a lethal challenge with NiV infection in Syrian hamsters. Remarkably, the nanoparticle vaccine stimulated the production of antibodies that exhibited superior cross-reactivity to homologous or heterologous *henipavirus*. These findings underscore the potential utility of ferritin-based nanoparticle vaccines in providing both broad-spectrum and long-term protection against NiV and emerging zoonotic *henipaviruses* challenges.

## Introduction

Nipah virus (NiV) is a highly lethal zoonotic paramyxovirus, belonging to the emerging *Henipavirus* genus, along with Hendra virus (HeV) (1). Since its first emergence in Malaysia in 1998, NiV outbreaks have become a near-annual occurrence in Bangladesh and India (2–4). The most recent outbreak of NiV occurred in the Indian state Kerala in August 2023 where six people have been infected, and two of whom have died since it emerged (5). NiV causes febrile encephalitis and severe respiratory disease in humans with a case-fatality rate (CFR) as high as 100% in some outbreaks (6). Fruit bats (*Pteropus* spp.), have been identified as the natural reservoir for NiV. During the initial Malaysian outbreak, pigs served as amplifying hosts through their consumption of contaminated fruit or waste products from infected fruit bats, though there was limited human-to-human transmission at that time (7). Conversely, the more recent outbreaks in India, Bangladesh, and the Philippines witnessed a significant role played by human-to-human transmission (4, 8–10). Furthermore, NiV exhibits a broad species tropism, which raises concerns about the potential for further outbreaks originating from infected livestock or domestic animals. The high CFR following NiV infection underscores the urgent need for the development of prophylactic or therapeutic medical countermeasures.

Due to the high mortality rates, the absence of effective medical countermeasures, and its potential for easy transmission, NiV is listed as a risk group 4 agent. The World Health Organization (WHO) has designated Nipah as a priority disease under the WHO Research and Development Blueprint (11). Based on the genetic characteristics, NiV can be divided into two main lineages, the Bangladesh and Malaysia lineages, responsible for outbreaks in different geographical regions (12, 13). The Malaysia strain (NiV-M) caused the initial outbreak in Malaysia and Singapore with a CFR of approximately 40%, and later caused an additional outbreak in the Philippines in 2014, with a CFR of around 52% (4, 14). Outbreaks of Bangladesh strain (NiV-B) have displayed a higher CFR of approximately 75%, with human-to-human transmission also observed (15). Previous research has shown that NiV-M and NiV-B exhibited notably different pathogenicity in African green monkeys (AGM) and Syrian hamsters. These differences in pathogenicity and ability for human transmission between NiV-M and NiV-B underscore the necessity for medical countermeasures capable of protecting against both the Bangladesh and Malaysia lineages (13, 16, 17).

Within the genus of *Henipavirus*, Hendra virus (HeV) shares similar pathological characteristics with Nipah virus (NiV) and has caused infections in humans in Australia (18). HeV primarily circulates among flying foxes and is known to be fatal to horses and humans. As of July 2022, a total of 66 natural HeV spillover events have been documented in horses in Australia, resulting in 105 horse fatalities (19, 20), along with 7 confirmed human cases, of which 4 resulted in fatalities (21). In the pursuit of treatment, the only human monoclonal antibody that has been evaluated for NiV protection studies in the African green monkey model and has undergone a phase I clinical study, m102.4 (22). Furthermore, a commercial equine HeV vaccine (Equivac^®^ HeV)) has recently been licensed in Australia and is currently in clinical development as an emergency vaccine countermeasure for potential Nipah virus outbreaks. Beyond HeV and NiV, other related *henipaviruses* have frequently emerged in China, including Langya and Mojiang viruses, which have been detected in individuals with febrile and pneumonic conditions (23, 24). Consequently, there is an urgent need to develop a comprehensive anti-*henipavirus* strategy aimed at mitigating outbreaks not only of both Nipah viruses but also potentially emerging zoonotic *henipaviruses*.

In this study, we presented the immunogenicity of a mosaic NiV G nanoparticle, which represents a divalent vaccine designed by covalently attaching NiV G proteins from both NiV-M and NiV-B to a 24-mer ferritin nanoparticle (25). This innovative mosaic NiV G nanoparticle and the divalent NiV G vaccine were subjected to thorough *in vitro* assessments of their immunogenicity and rigorous *in vivo* evaluations of their protective efficacy. Our data clearly demonstrated that, when compared to the conventional NiV G subunit vaccine, the mosaic nanoparticle vaccine elicited significantly higher and more enduring immune responses against both NiV-M and NiV-B. Furthermore, our findings indicate that the nanoparticle vaccine offers highly effective protection against NiV infection, particularly in the Syrian hamster model. Notably, the nanoparticle vaccine also displayed improved cross-reactivity against other related *henipaviruses*. These promising results underscores the potential of the mosaic nanoparticle approach to induce broader and more comprehensive antibody responses compared to traditional subunit vaccine. If further validated in additional preclinical models and human clinical trials, this approach could represent a valuable addition to the strategies available for combating the outbreaks of *henipaviruses*.

## Results

### Development and characterization of divalent mosaic NiV-G ferritin-based nanoparticle vaccines

**Figure 1:**
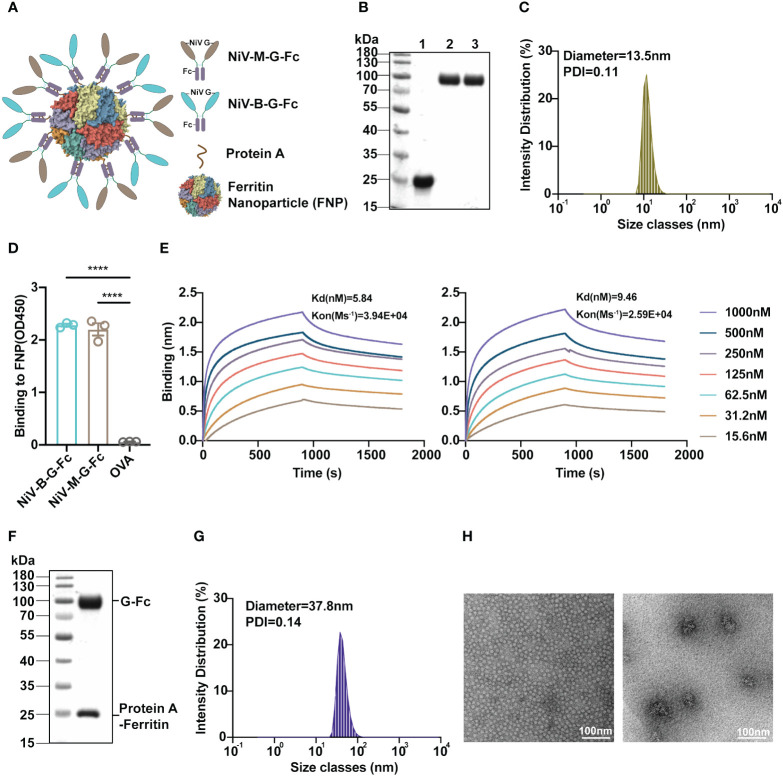
Development and characterization of the FNP-NiV G vaccine. **(A)** The schematic representation illustrates the G proteins of both NiV-M (in dark orange) and NiV-B (in light blue), each fused with an Fc tag (in purple), a ferritin-based 24-meric nanoparticle with N-terminal protein A tag (in bronze), and an FNP-NiV G complex. **(B)** Purifications of Ferritin-protein A-His (lane 1) and NiV-G-Fc proteins (lane 2: NiV-M-G-Fc, lane 3: NiV-B-G-Fc) following size exclusion chromatography (SEC) were analyzed by SDS-PAGE. **(C)** Dynamic light scattering (DLS) determined the size distribution of the FNP complex. **(D)** The binding affinity of FNP to NiV-M-G-Fc or NiV-B-G-Fc was assessed using ELISA. Equal amounts of both NiV-G-Fc proteins were coated on ELISA plate with OVA serving as a negative control. Subsequently, FNP fractions were added. Binding of FNP to NiV-G-Fc was detected by anti-His tag antibody. Data are presented as mean ± SEM (n=4), and statistical significance was determined through a Student’s two-tailed t-test. ***p < 0.0001. **(E)** The interaction between FNP and NiV-G-Fc (NiV-M, left) or NiV-G-Fc (NiV-B, right) was quantified using Bio-Layer Interferometry (BLI). Experiments were performed three times with similar results, and one set of representative data is displayed. **(F)** SDS-PAGE analysis of the FNP-Fc-NiV G complex after size exclusive chromatography (SEC). **(G)** The size distribution of the FNP-NiV G complex was determined by DLS. **(H)** Negative-stain electron microscopy (EM) analysis of FNP (left) and FNP-NiV G complex (right). Scale bar = 100 nm. .

Nipah virus isolates from Malaysia (NiV-M) and Bangladesh (NiV-B) have been responsible for recurrent outbreaks in their respective regions. To address the challenge of developing a vaccine effective against both strains, we have designed a mosaic divalent virus-like nanoparticle (VLP) system presenting both the NiV-M and NiV-B G proteins on its surface. In this system, we separately constructed the ferritin (containing an N-terminal protein A tag) (His-Ferritin-protein A) and NiV G proteins fused with a C-terminal Fc tag (NiV G-Fc) (**Figure 1A**). Twenty-four copies of ferritin could form a structural scaffold, while the NiV G (residues 176 aa-602 aa) served as essential immunogens (**Figure 1A**). The purified NiV G-Fc proteins spontaneously form nanoparticles through the conjugation of Fc tag and protein A (**Figure 1A**). This unique mechanism enables the assembly of antigens derived from both NiV-M and NiV-B isolates onto the ferritin nanoparticle (FNP) through independent protein preparations and subsequent protein A-Fc mediated VLP formation.

His-Ferritin-protein A was initially expressed and purified from *Escherichia coli* using His tag chromatography (**Figure 1B**). Subsequent size exclusive chromatography analysis revealed that His-Ferritin-protein A could spontaneously self-assembled into a large VLP nanoparticle (FNP), as indicated by its elution profile (**Supplementary Figure 1A**). Further characterization using dynamic light scattering (DLS) and negative-stained electron microscopy (EM) confirmed the spherical nature of the nanoparticles, with an average diameter of approximately 13.5 nm (**Figures 1C, H**), consistent with the 24-meric VLP particle observed in previous EM studies (25). Additionally, Fc tagged NiV G proteins (NiV-M-G-Fc and NiV-B-G-Fc) were successfully expressed and purified from mammalian cells (HEK293F) with high homogeneity, as demonstrated by their size exclusive chromatography profiles (**Figure 1B** and **Supplementary Figures 1B, C**). Consequently, both ferritin-based nanoparticle and Fc tagged NiV G proteins were prepared effectively for vaccine construction.

To prepare the NiV VLP vaccine (FNP-NiV G), we initially investigated the binding between the protein A-tagged FNP and Fc-tagged NiV G using both enzyme-linked immunosorbent assay (ELISA) and Bio-Layer Interferometry (BLI). Both NiV-M-G-Fc and NiV-B-G-Fc demonstrated strong binding affinity to FNP (**Figures 1D, E**). The binding affinity of NiV-M-G-Fc and NiV-B-G-Fc for the NiV cell receptor, EphrinB2, was then evaluated by ELISA (**Supplementary Figure 2A**), flow cytometry (**Supplementary Figure 2B**) and BLI (**Supplementary Figures 2C, D**) in a dose-dependent manner, respectively. The FNP-NiV G vaccine allows the presentation of 24 copies of Fc-tagged dimeric NiV G, corresponding to 48 copies of NiV G, on the surface of the 24-meric protein A-tagged FNP. Subsequently, we conducted the assembly of NiV-M-G-Fc and NiV-B-G-Fc onto the 24-meric FNP by mixing them in a molar ratio of 24:24:1. The FNP-NiV G complex was successfully co-eluted and co-purified through size-exclusive chromatography. The eluted fractions were analyzed by SDS-PAGE and pooled together (**Figure 1F** and **Supplementary Figure 1D**). Furthermore, the FNP-NiV G complex was evaluated by DLS and negative EM, confirming the spherical shape of the nanoparticle at approximately 37.8 nm but with a fuzzy surface (**Figures 1G, H**).

### FNP-NiV G vaccine induced higher level of humoral responses in mice

**Figure 2:**
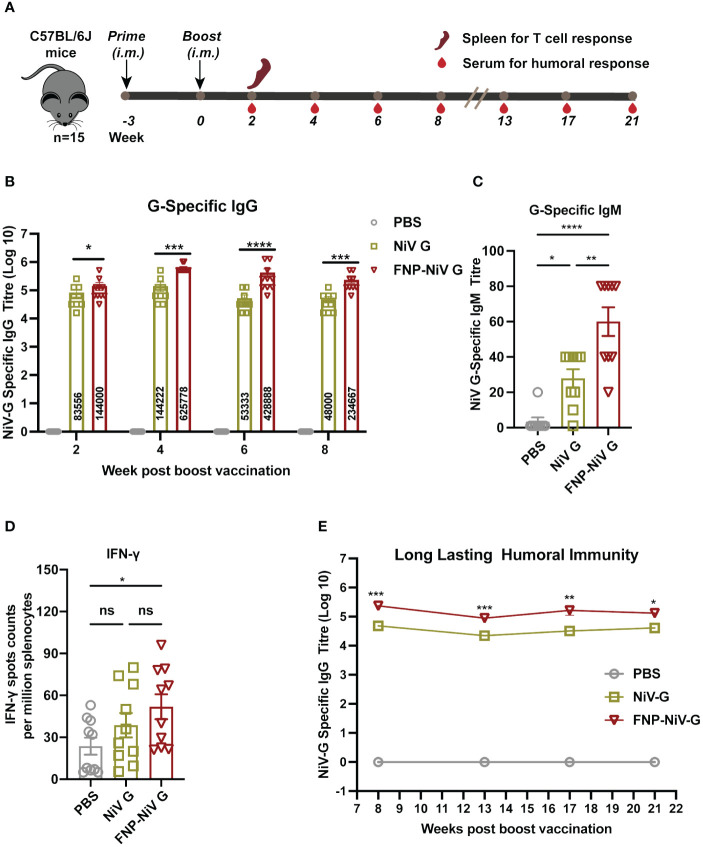
Vaccination schedule and immune responses induced by vaccine regimens in mice. **(A)** All mice were primed and boosted 3 weeks later with the indicated vaccines via intramuscular injection (i.m.) in a volume of 50 μL. Mouse sera were collected for assessment of NiV G-specific antibody titers at indicated time points post boost vaccination. To evaluate T cell memory responses, spleen was collected 2 weeks after the boost. **(B)** NiV G-specific IgG antibodies in collected mice sera were detected using ELISA. In the ELISA assay, plates were pre-coated with recombinant NiV G, and antibody titers were reported as the highest serum dilution that remained detectable (defined as signal being at 2.1-fold of the blank). The data are presented as mean ± SEM (n = 10 for each mouse group). **(C)** Mouse sera were collected two weeks post boost immunization for detection of G-specific IgM antibodies using ELISA. **(D)** Splenocytes were stimulated with the peptides scanning the G protein and the IFN-γ secretion in splenocytes were detected by an ELISpot assay two weeks post boost immunization. Data represented as mean ± SEM (n = 5). **(E)** Long-lasting humoral immunity up to 21 weeks after boost vaccination was detected by ELISA. The data are presented as mean ± SEM (n = 10 for each mouse group). Statistical differences among the groups were analyzed using a Student’s two-tailed t-test. ***p < 0.001; **p < 0.01; *p < 0.05; ns, not significant.

We proceeded to assess the effectiveness of the FNP-NiV G vaccine in eliciting neutralizing antibody responses against NiV, comparing it with a subunit vaccine based on NiV G. To this end, we immunized C57BL/6J mice with FNP-Fc-NiV G (FNP-NiV G) and NiV G-Fc (NiV G), respectively. Three weeks after the primary immunization, the mice received a booster dose. Mouse sera were collected at weeks 2, 4, 6 and 8 following the boost immunization for up to 21 weeks (**Figure 2A**). These sera were analyzed to assess their antibody titers and neutralization against two NiV isolates. First, the specific IgG titer against NiV G protein induced by FNP-NiV G was significantly higher than that induced by NiV G subunit vaccine from week 2 post the boost vaccination to week 8 (**Figure 2B**). Second, the FNP-NiV G vaccine induced higher levels of NiV G-specific IgM antibodies at week 2 post the boost vaccination, compared to the NiV G vaccine, indicating its potential to provide rapid protective immunity (**Figure 2C**). Third, we assessed the cellular immune responses in mice immunized with FNP-NiV G and NiV G vaccines. Splenocytes were isolated from both immunized (FNP-NiV G and NiV G groups) and control mice (PBS group) 2 weeks after the booster dose and then stimulated *in vitro* with NiV G protein peptides. The specific spots indicating the presence of IFN-γ were more numerous in the FNP-NiV G immunized animals than in the NiV G group, suggesting Th1-biased cellular immune response, albeit with a modest increase (**Figure 2D**). To evaluate the long-term duration of immunity by FNP-NiV G vaccination, we measured the NiV G-specific IgG level in the sera of immunized mice at weeks 13, 17 and 21 after the boost vaccination. Despite a decline in IgG titer compared to week 4, the humoral immunity induced by both nanoparticle (FNP-NiV G) and subunit vaccine (NiV G) maintained high and stable levels for several months (**Figure 2E**). Notably, the nanoparticle vaccine exhibited significantly higher anti-NiV IgG levels, suggesting its effectiveness in providing long-term protection against NiV. These findings underscore the potency of the FNP-NiV G vaccine in generating robust and long-lasting immune responses against Nipah virus.

### Enhanced neutralizing activity of FNP-NiV G vaccine against NiV in mice

**Figure 3:**
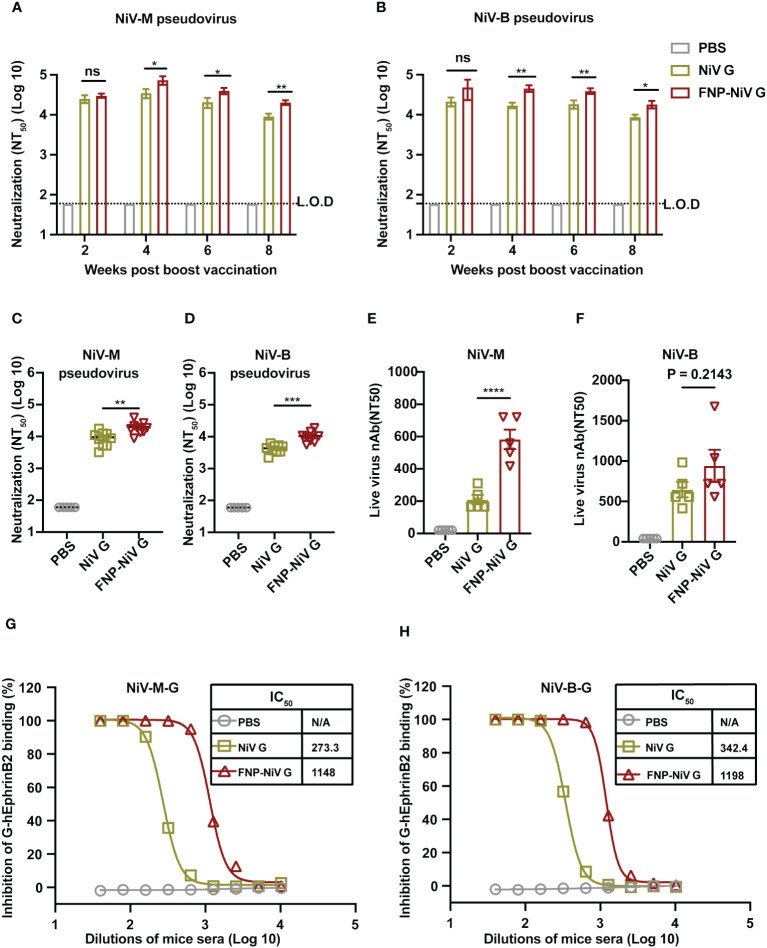
Neutralizing activity of FNP-NiV G vaccine against NiV-M and NiV-B. **(A, B)** Mouse sera at indicated timepoints were examined for neutralizing antibodies against cell entry of pseudotyped NiV of the Malaysia stain (NiV-M) **(A)** and the Bangladesh strain (NiV-B) **(B)**. **(C, D)** Long-term neutralizing activity of subunit NiV G and FNP-NiV G vaccines was analyzed. Mouse sera at week 21 post-boost immunization were examined for neutralizing antibodies against the cell entry of pseudotyped NiV-M **(C)** and NiV-B **(D)**. **(E, F)** Mouse sera collected at week 4 post boost-immunization were also examined for neutralizing antibodies against cell infection of authentic NiV-M **(E)** and NiV-B **(F)**. **(G, H)** The inhibitory potency of immunized sera on NiV-EphrinB2 binding in hEphrinB2-Raji cells was evaluated. Mouse sera from week 4 post-boost immunization were used to block the interaction between the human EphrinB2 receptor and NiV-M G **(F)** or NiV-B G **(G)** protein using flow cytometry. Recombinant G protein was incubated with cells expressing hEphrinB2 in the presence of serially diluted mouse sera, and the efficiency of binding was characterized by the flow cytometry signal (i.e., fluorescence intensity of cells). Inhibition (%) was calculated from the flow cytometry signal in the presence or absence of mouse sera. The data were presented as mean ± SEM (n = 10 for mice in each group). Experiments were performed three times, statistical differences among the groups were analyzed using a Student’s two-tailed t-test. L.O.D. represents the limit of detection.

The neutralizing efficacy of sera from immunized mice was evaluated against both pseudotyped and authentic Nipah viruses. Mouse sera collected at weeks 2, 4, 6 and 8 post the boost immunization were analyzed for their potency in neutralizing the cell entry of both NiV-M and NiV-B. Additionally, to determine the competence and duration of immune protection conferred by FNP-NiV G vaccination, we measured the neutralizing activity in the sera of FNP-NiV G-immunized mice on week 21, exceeding five months post the booster vaccination. Firstly, the peak neutralizing antibody levels were observed on week 4, with a gradual reduction thereafter (**Figures 3A, B**). Secondly, sera from FNP-NiV G-immunized mice exhibited superior neutralizing activity compared to those from mice solely immunized with NiV G, effectively neutralizing both NiV-M and NiV-B pseudoviruses (**Figures 3A, B**). Thirdly, sera collected on week 21, more than 5 months post boost immunization, maintained efficient and similar neutralization against NiV-M and NiV-B pseudoviruses compared to week 8, indicating prolonged immune protection (**Figures 3C, D**). Fourthly, FNP-NiV G-induced sera displayed more potent neutralization of both authentic NiV (NiV-M and NiV-B) infection compared to the NiV G vaccine (**Figures 3E, F**). Taken together, these results collectively demonstrate that, in comparison to the subunit vaccine, the nanoparticle vaccine induces higher-titer neutralizing antibody responses and more effectively inhibits the infection of two genetically distinct NiV isolates. Furthermore, these neutralizing antibody exhibit relatively long-term persistence, indicating the potential of the nanoparticle vaccine to provide sustained immune protection in animals.

To understand the mechanism of vaccine-induced antibodies neutralize NiV infection, we further investigated the interactions between NiV G and human EphrinB2 (hEphrinB2) in the presence of sera from immunized mice. To this end, the flow cytometry assay was conducted, where recombinant NiV G protein of both NiV-M and NiV-B were incubated with cell-surface expressed hEphrinB2, in the presence of mouse sera from either the NiV G vaccine or FNP-NiV G vaccine. Notably, antibodies induced by both vaccines effectively hindered the binding of NiV-M and NiV-B G proteins to hEphrinB2 in a dose-dependent manner, with the antibodies induced by the FNP-NiV G vaccine exhibiting much greater potency (**Figures 3G, H** and **Supplementary Figure 3**). These findings underscore that, in comparison to the subunit vaccine, the nanoparticle vaccine elicits antibodies of significantly higher titers, capable of blocking NiV G binding to hEphrinB2 and effectively neutralizing NiV infection of target cells.

### Complete protection provided by nanoparticle vaccine in Syrian hamster against NiV challenge

**Figure 4:**
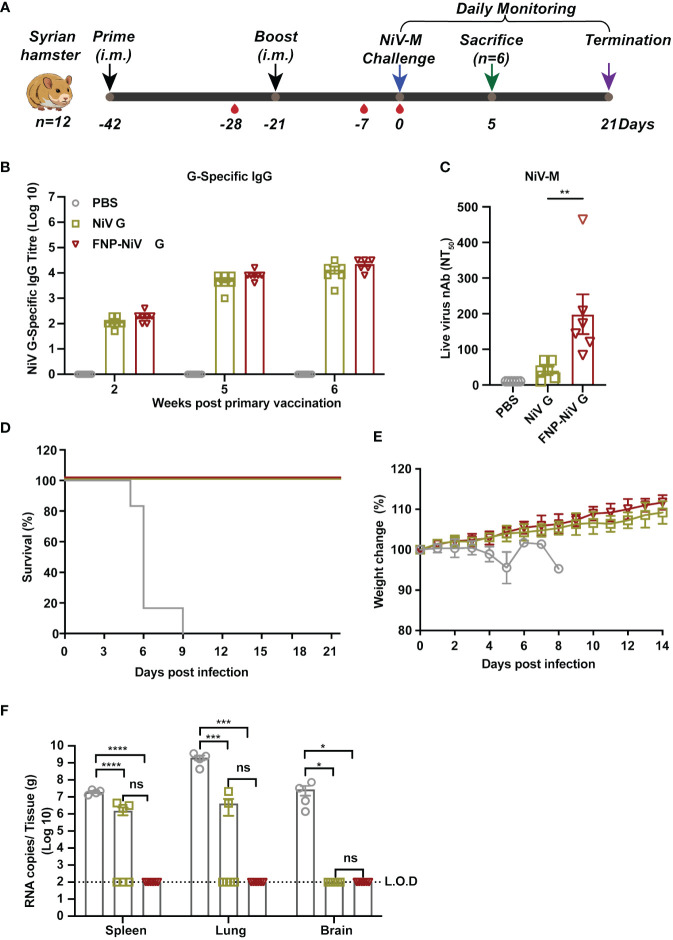
The protection of FNP-NiV G vaccine in Syrian hamsters challenged with NiV. **(A)** Immunization schedule of vaccine regimens and virus challenge in Syrian hamsters. All hamsters were primed and boosted in a three-week interval with indicated vaccines via intramuscular injection (i.m.) in a volume of 100 μL. Serum was collected to assess NiV G-specific antibody titers at indicated time points post-vaccination. On week 3 post-boost immunization, hamsters were infected with NiV-M. Daily monitoring of hamsters for weight change and survival was conducted for three weeks post-challenge. At day 5 post-infection, half of the hamsters were sacrificed for viral load detection and histopathological analysis. **(B)** Analysis of specific IgG antibodies against the NiV G protein induced by the vaccine regimens using ELISA. **(C)** Measurement of the level of neutralizing antibodies in hamster sera induced by different vaccine regimens using a neutralization assay against NiV-M. **(D, E)** Survival **(D)** and weight loss **(E)** of Syrian hamsters challenged with NiV-M. **(F)** Viral burden in spleen, lung, and brain tissues collected from challenged hamsters at 5 dpi was determined by Real-time RT-PCR. The data were presented as mean ± SEM (n = 6 for hamsters in each group, 2 hamsters in the PBS group died at 4 dpi, and the remaining 4 were analyzed at 5 dpi). Statistical differences among the groups were analyzed using a Student’s two-tailed t-test.

To further explore the *in vivo* protection efficacy of FNP-NiV G against NiV challenge, the Syrian hamster model (n=6) was immunized with either the NiV G or FNP-NiV G vaccine at a dose of 10 μg. Three weeks post the booster immunization, hamsters were exposed to a lethal dosage (1000 LD_50_) of NiV-M strain via intraperitoneal inoculation, as illustrated in **Figure 4A**. This hamster model, mirroring the respiratory and neurological pathology seen in human cases with NiV infection, stands as a standard for pre-clinical NiV vaccine development (26). Here clinical disease assessment involved monitoring hamsters for survival and weight changes, while viral load in spleen, lung and brain tissues was measured using real time RT-PCR. Spleen, lung or brain pathology was evaluated by scoring tissue for histopathological changes. IgG titer and virus-neutralizing antibodies in sera before viral challenge were measured targeting NiV-M/NiV-B pseudoviruses and authentic virus (NiV-M). Consistent with results observed in mice, FNP-NiV G vaccination induced higher IgG titers and stronger neutralizing activity against NiV infection in hamsters (**Figures 4B, C** and **Supplementary Figure 4**). In contrast, no NiV G-specific IgG and neutralizing antibodies could be detected in serum obtained from the sham vaccinated group (PBS group) (**Figures 4B, C** and **Supplementary Figure 4**). Following inoculation with NiV-M, both NiV G and FNP-NiV G vaccinated hamsters survived the challenge, whereas all animals in the sham-vaccinated group exhibited gross weight loss from day 4 post infection and succumbed to respiratory diseases (**Figures 4D, E**). Moreover, in comparison to NiV G vaccinated group, hamsters of FNP-NiV G vaccinated group demonstrated slightly more robust weight recovery, indicating that FNP-NiV G provided superior protection against NiV infection (**Figure 4E**). The results of viral load detection below corroborated these findings (**Figure 4F**). Firstly, the sham vaccinated hamsters contained high viral burden in the spleen (average viral load ± SEM = 2.0×10^7^ ± 3.0×10^6^ RNA copies/g), lung (2.0×10^9^ ± 6.7×10^8^ RNA copies/g), and brain tissues (2.8×10^7^ ± 1.6×10^7^ RNA copies/g) (**Figure 4F**). Secondly, viral RNA was detected in spleens of three hamsters (average viral load ± SEM = 1.6×10^6^ ± 7.8×10^5^ RNA copies/g), and lungs of two hamsters (4.2×10^6^ ± 3.4×10^6^ RNA copies/g), and their brains are free of viral RNA, in six hamsters of NiV G vaccinated group (**Figure 4F**). The viral burden in infected spleen or lungs was significantly lower than that in the PBS group (**Figure 4F**), suggesting the NiV subunit vaccine provided most but not complete protection against NiV-M challenge (l000 LD_50_) despite all animals survived (**Figure 4D**). Thirdly, it is worth noting that no virus was detected in all the dissected tissues (spleen, lung and brains) of hamsters immunized with FNP-NiV G vaccine (**Figure 4F**).

**Figure 5:**
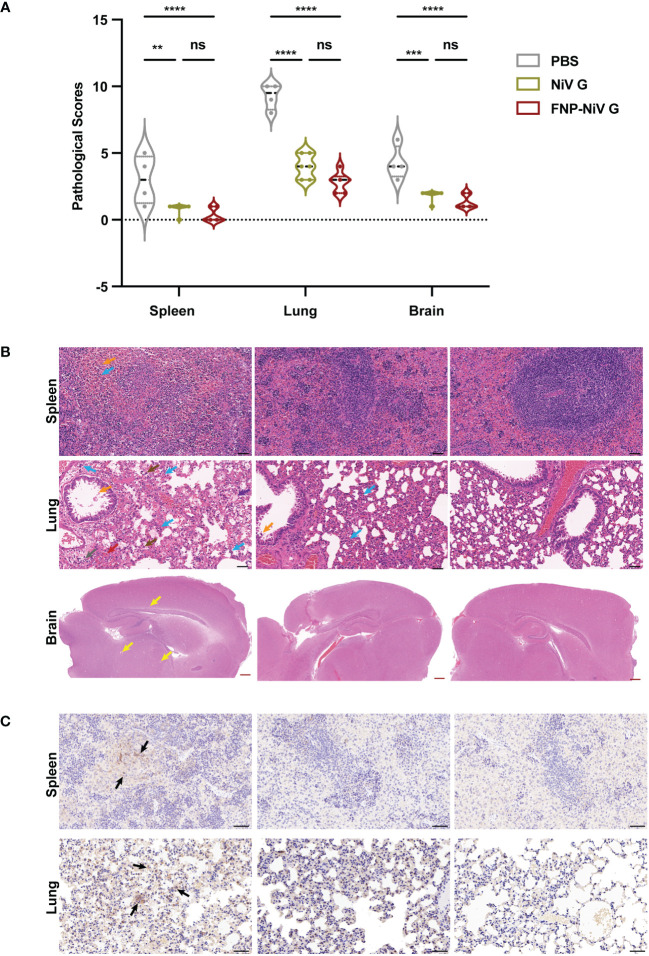
Pathological changes in tissues of hamsters challenged with NiV-M at 5dpi. **(A)** Scoring of tissue pathological damage in hamsters vaccinated with PBS, NiV G and FNP-NiV G after NiV-M challenge. The pathological score reflects the severity of tissue damage, with scoring details corresponding to **Supplementary Table 1** ((n = 6 for hamsters in each group, 4 hamsters remaining for PBS group). Statistical differences among the groups were analyzed using a Student’s two-tailed t-test. **(B)** Hematoxylin and eosin staining (HE) of spleen, lung, and brain sections. In the PBS group, the spleen exhibited loss of normal splenic architecture, lymphocyte necrosis, lymphocyte infiltration (blue arrow), and hemorrhage (orange arrow). No pathology was observed in vaccinated animals. The lungs of PBS hamsters showed pronounced bronchointerstitial pneumonia signs, including multifocal alveolar hemorrhage (red arrow), extensive lymphocyte infiltration (blue arrow), mononuclear macrophages (brown arrow), thickening of alveolar walls, collapsed alveoli, eosinophilic secretion in small bronchial lumens (orange arrow), alveolar edema (purple arrow), and perivascular edema (gray arrow). The lungs of vaccinated hamsters displayed no significant histopathological abnormalities. Perivascular edema (yellow arrows) was present in the brains of the PBS group, with fewer or no signs observed in vaccinated hamsters. The black and red scale bars indicate 50 μm and 500 μm, respectively. **(C)** Representative immunohistochemistry (IHC) of spleen and lung tissues with NiV N-specific antibodies. Immunostaining was indicated in dark brown (black arrows). Scale bar =50 μm.

Spleen, lung and brain tissues harvested at 5 days post infection (5 dpi) were then evaluated for pathological changes by HE staining and IHC analysis. Pathological changes of tissues were evaluated by scoring following the principle of 4-point scoring system (27) (**Supplementary Table 1**). The number of scores indicated the degree of severity, where 0 indicates no change or within normal limits, 1 to 4 represents the minimal, slight, moderate and severe histopathological changes. Consistent with the results of viral RNA detections, the histopathological scores of tissues from PBS group are higher than that of the other groups (NiV G and FNP-NiV G) (**Figure 5A**). Hamsters in PBS group developed gross and obvious histopathological injures in spleen, lung and brain, whereas hamsters in NiV G or FNP-NiV G vaccinated groups exhibited much fewer or no histopathological changes (**Figures 5A, B** and **Supplementary Table 1**). Specifically, without vaccine protection, the spleens exhibited single-cell necrosis or focal necrosis, infiltration of lymphocytes and hemorrhage, accompanied with untidy margin between the white pulps and red pulps (**Figure 5B** and **Supplementary Figure 5**). By contrast, the hamsters from NiV and FNP-NiV G vaccinated groups maintained almost normal tissue structures with no obvious pathological damage, albeit with minimal degree of infiltration of lymphocytes (**Figure 5B** and **Supplementary Figure 5** and **Supplementary Table 1**). As reported, the clinical signs of NiV infections primarily focus on the respiratory system. The lungs of hamsters with NiV-M infection manifested with increased thickness of alveolar walls, hemorrhage, infiltration of lymphocytes and edema fluid surrounding vascular and alveolar walls, which were observed obvious in hamsters of PBS group but alleviate or disappeared in NiV G and FNP-NiV G vaccinated groups (**Figure 5B** and **Supplementary Table 1**). Post lethal infection of NiV-M in Syrian hamsters, the histopathological changes in brains of the PBS group were not obvious, only with mild congestion or perivascular edema fluid and very few infiltrations of lymphocytes, but still with significantly higher pathological scores than the vaccinated groups. (**Figure 5B** and **Supplementary Table 1**). Additionally, IHC analysis with NiV-N protein specific antibody confirmed the proliferation of NiV in spleen and lung tissues of hamsters in PBS-vaccinated group, while the block of virus replication for either NiV G or FNP-NiV G groups (**Figure 5C**), which was consistent with the results in viral burden detection (**Figure 4F**). These outcomes demonstrated that the FNP-NiV G vaccine provided complete protection for hamsters against NiV challenge, surpassing the efficacy of the NiV G vaccine.

### Broad cross-reactive immune responses elicited by FNP-NiV G vaccine against other *henipaviruses*

**Figure 6:**
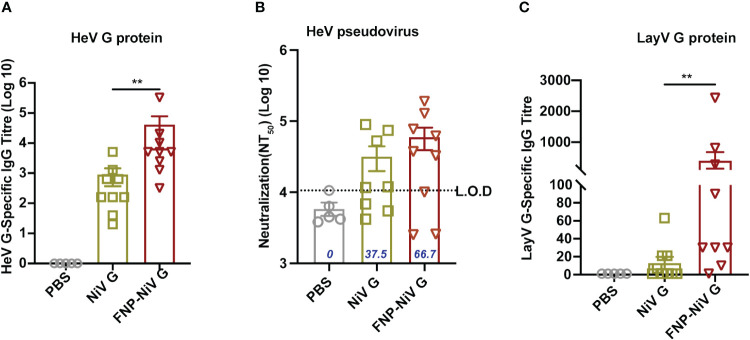
Cross-reactive responses elicited by FNP-NiV G vaccine. **(A)** HeV G-specific IgG antibodies in mice sera at week 7 were detected by ELISA with HeV G protein as an antigen coated on ELISA plates. **(B)** Levels of cross-neutralizing antibodies from FNP-NiV G vaccinated mice were measured using pseudotyped HeV. The percentage (%) of sera samples exhibiting neutralizing activity in each group was labelled out. **(C)** Langya virus (LayV) G protein-specific antibodies were detected by LayV G-based ELISA assay. The data were presented as mean ± SEM. Statistical differences among the groups were analyzed using a Student’s two-tailed t-test.

To assess the cross-reactivity of NiV G subunit vaccine and FNP-NiV G vaccine with other *henipaviruses*, including Hendra Virus (HeV) and recently emerging Langya Virus (LayV), mouse sera post-booster immunization were analyzed for HeV or LayV G protein- specific IgG titers and neutralization activity against HeV or LayV. Purified HeV and LayV G proteins were coated as antigen to detect the cross-reactive IgG in sera collected on week 4. In comparison to PBS control group, both the NiV G and FNP-NiV G vaccines could induced cross-reactive antibodies to HeV G, with the sera from FNP-NiV G vaccinated hamsters exhibiting a significantly elevated cross-reactive IgG titer (**Figure 6A**). Furthermore, pseudovirus neutralization assays were conducted to assess the level of neutralizing antibodies against HeV induced by NiV G and FNP-NiV G vaccines. The nanoparticle vaccine demonstrated an increased cross-neutralizing capability against HeV pseudovirus, suggesting enhanced cross-protection against HeV infection (**Figure 6B**). Notably, regarding the recently emerged Langya virus (LayV), another member within *Henipavirus* genus, immunization of the FNP-NiV G elicited antibodies with a significantly elevated cross-reactivity to LayV G protein, in comparison to NiV G subunit vaccine (**Figure 6C**). Regrettably, the assessment of cross-reactive neutralization against LayV could not be conducted in this study due to the limited availability of LayV pseudovirus or authentic virus.

## Discussions

As an emerging zoonotic pathogen, the Nipah virus has triggered severe epidemic outbreaks, characterized by a high case fatality rate, thereby presenting a persistent threat to global human health (28). The imperative to proactively address this ongoing risk has underscored the critical need for a safe and effective vaccine to prevent the NiV infection and transmission. The development of such a vaccine is crucial as a robust countermeasure, especially in light of the urgency to prepare for potential future outbreaks. Despite active efforts in vaccine development against NiV, none have yet reached commercial availability for this lethal disease.

*Henipavirus* vaccines in development primarily target the surface G glycoprotein or the fusion F protein. A diverse array of vaccine candidates has been explored across various animal models, demonstrating varying levels of protection. Currently, three types are either in or have completed clinical stages. Among these, the most promising vaccine against both NiV and HeV is the HeV soluble G protein-based subunit vaccine (HeV-sG). Extensive testing across species, including cats, ferrets, African green monkeys (AGM), and horses, has shown complete protection against lethal challenges from NiV-M, NiV-B, or HeV (29–36). As of May 6, 2024, the Phase I clinical study for HeV-sG has been concluded, although it has not yet received approval and licensure. Another vaccine, mRNA-1215, utilizes a unique approach by combining the secreted prefusion-stabilized F covalently linked to G (pre-F/G) from the Malaysia strain NiV, resulting in post-expression trimerization (37, 38). mRNA-1215 vaccine is currently undergoing evaluation in a Phase I clinical trial for NiV (39). The third vaccine in Phase I clinical trial is a recombinant vesicular stomatitis virus (VSV) vectored vaccine expressing the glycoprotein from the Bangladesh strain (rVSV-ΔG-NiV-B G), with ongoing evaluations in AGM, and phase I clinical trials in progress as well (40). Additionally, various types of NiV vaccines targeting the G and F protein, including viral vector vaccines (41–44) and DNA vaccines (43, 44) are under preliminary development. It is premature to determine whether any of these candidate vaccines will secure licensure and meet the preferred product characteristics outlined in the draft WHO target product profile for a NiV vaccine (45).

Subunit vaccines, lacking infectious viral components, are generally considered safer than virus-based vaccines, though some may exhibit lower immunogenicity. Historical vaccine research has predominantly targeted specific strains of NiV or HeV. However, the occurrence that distinct pathogenic strains of NiV responsible for human outbreaks in various geographic regions suggests that current vaccine designs focused on single antigens may not sufficiently elicit protective antibodies against these variants (17). Thus, exploring the potential of multivalent antigens in vaccine candidates against concerning strains is warranted. *Henipavirus* attachment glycoproteins (G), pivotal in the initial step of viral infection by attaching to host cells, are highly immunogenic among *henipavirus* proteins, making them prime candidates for subunit vaccine design. In our study, we employed a virus-like nanoparticle approach to enhance the neutralizing immunogenicity of NiV G as a subunit vaccine. More notably, G protein derived from two strains of NiV (NiV-M and NiV-B) were presented on the nanoparticle surface simultaneously. The method involved preparing a ferritin protein-based nanoparticle that spontaneously assembled into a 24-mer virus-like particle (VLP). This VLP was N-terminally tagged with 24 copies of protein A on the surface. Subsequently, divalent Fc-tagged dimeric G proteins of both NiV-M and NiV-B were prepared. Upon conjugating the protein A-tagged VLP nanoparticle with the Fc-tagged G, the resulting assembled nanoparticle could present 48 copies of divalent G proteins on its surface. Biolayer Interferometry analysis revealed that G proteins of NiV-M and NiV-B could efficiently bound to the ferritin-based nanoparticle, albeit a slightly different binding affinity. In comparison to current subunit vaccines, this divalent VLP nanoparticle, with its high local density, mimics G proteins on virus particles, potentially inducing more potent, enduring immune responses and offering improved protection against various NiV variants or *henipaviruses* infections (46, 47). Furthermore, nanoparticle virus-like particle (VLP) vaccines stand out as a highly promising and versatile immunization strategy, offering an exceptional combination of safety, immunogenicity, stability, and versatility when compared to other types of vaccines (48–50). Based on the presented results in this study, our VLP-based NiV G vaccine design holds promise for a novel NiV vaccine with significantly enhanced and broader cross-protective neutralizing immunogenicity. It is worth noting that the viral challenge assay conducted in Syrian hamsters specifically targeted the NiV Malaysia strain. Further studies are planned to test its protection efficacy against the Bangladesh strain and even Hendra viruses. Moreover, the number of immunization times, vaccination routes and doses will be optimized to enhance its efficacy, accessibility, and efficiency.

In summary, this study highlights the effectiveness of immunization with a self-assembling ferritin-based nanoparticle vaccine in eliciting a robust humoral immune response against two genetically distinct strains of NiV. The FNP-NiV G vaccine demonstrates potent and broad-spectrum neutralizing efficacy against both the Malaysia and Bangladesh strains of NiV, as well as HeV or LayV. Furthermore, it exhibits superior and complete protection against live virus challenge in Syrian hamsters in comparison to NiV G subunit vaccine. This study holds significant promise for facilitating rapid response to emerging NiV outbreaks, and future investigations should explore live virus challenge studies with additional NiV stains and other related *henipaviruses* when conditions permit.

## Materials and methods

### Ethics statement

All animal work was performed in strict accordance with the guidance and recommendations in the Guide for the Institutional Animal Care and Use of Laboratory Animals. Experiments were conducted under animal use protocols approved by the Institutional Animal Care and Use Committee (Ethics Number: WIVAF42202201) of the Wuhan Institute of Virology, Chinese Academy of Sciences.

All live NiV infection was performed under BSL-4 conditions at the National Biosafety Laboratory (Wuhan), Chinese Academy of Sciences, under the standard operating procedure approved by the Institutional Biosafety Committee.

### Cell lines, plasmids and viruses

HEK293T cells (ATCC, CRL-3216) and Vero cells (ATCC, CCL-81) were cultured in Dulbecco’s modified Eagle medium (DMEM) (Gibco, NY, USA) supplemented with 10% fetal bovine serum (FBS) (Gibico) and 50 U/mL penicillin/streptomycin (Gibco) at 37°C. Raji cells (ATCC, CCL-86) were maintained in RPMI 1640 medium (Gibco) supplemented with 10% FBS (Gibco) and 50 U/mL penicillin/streptomycin (Gibco) at 37°C. HEK293F cells were cultured with shaking at 37°C and 8.0% CO_2_ in FreeStyle 293 Expression Medium (Gibco). BL21 (DE3) *E. coli* cells obtained from TransGen Biotech (Cat. No. CD601–02) were cultured in Luria-Bertani (LB) medium containing 50 μg/mL Kanamycin.

The genes encoding the extracellular domain of Nipah virus attachment glycoprotein (NiV G, residues 176 aa-602 aa) of NiV-M (GenBank Accession: MK673562.1) and NiV-B (GenBank Accession: MK673565.1) isolates, *Helicobacter pylori* ferritin (NCBI Reference Sequence: WP_000949190.1), and domain B of *S. aureus* protein A (residues 212 aa-270 aa) (NCBI Reference Sequence: WP_190282922.1) were codon-optimized and synthesized by GenScript. The NiV G ectodomain (residues 176 aa-602 aa) was subcloned into pcDNA3.4-hIgG1-Fc vector (pcDNA3.4-NiV G-hIgG1-Fc) and pcDNA3.4 vector with a C-terminal 6×His tag (pcDNA3.4-NiV G-His). Ferritin was subcloned into the pET-28a (+) vector with an N-terminal domain B of protein A and 8×His tag (pET-28a-His-protein A-Ferritin). For NiV pseudoviruses (NiVpp) production, the genes of attachment protein(G) and fusion protein (F) from NiV-M and NiV-B were codon-optimized and cloned into eukaryotic expression plasmid pcDNA3.1 to generate the recombinant plasmids. A luciferase-expressing HIV-1 genome plasmid (pNL4–3.luc.R-E-) was maintained in our laboratory.

The cDNA encoding human EphrinB2 containing a C-terminal Flag tag was codon-optimized, synthesized and inserted into the lentivirus vector pLVX-IRES-mCherry vectors (pLVX-IRES-mCherry-EphrinB2-Flag). psPAX2 (Addgene#12260) and pMD2.G (Addgene #12259) were also maintained in our laboratory.

The Nipah virus isolates of Malaysia (NiV-M) and Bangladesh (NiV-B) were obtained from the National Virus Resource Center (NVRC), Chinese Academy of Sciences. The NVRC Accession Number was listed in **Supplementary Table 2.**

### Protein expression and purification

Proteins related to NiV G were expressed in HEK293F cells. Briefly, pcDNA3.4-NiV G-hIgG1-Fc or pcDNA3.4-NiV G-His plasmids were transfected into HEK293F cells using PEI Transfection Reagents (Polysciences, 23966). Five days post transfection, supernatant containing the Fc-tagged NiV G (NiV G-Fc) or His-tagged NiV G (NiV G-His) proteins were collected and purified using Protein A agarose (Beyotime, P2015) or Ni Sepharose (Cytiva, 17526801), respectively. The his-tagged ferritin-based nanoparticle (FNP) was expressed in BL21 (DE3) *E. coli* cells. Protein expression was induced using IPTG (isopropyl-beta-D-thiogalactoside) at a final concentration of 1mM at 37°C and purified using Ni Sepharose as well. These purified proteins were further subjected to Superose 6 Increase 10/300 GL gel filtration chromatography. The purified FNP and NiV G-Fc proteins were co-incubated (molar ratio is 1:24) at room temperature for 1 hour. Subsequently, the formed complex was purified using gel filtration chromatography. The diameters of FNP and FNP-NiV G were characterized using dynamic light scatter (DLS, Wyatt Technology), and the purified proteins were analyzed by SDS-PAGE.

### BLI binding assays

The BLI experiments were performed in PBS (pH8.5) supplemented with 0.1% BSA and 0.02% Tween-20 using the Octet RED instrument (Sartorius). His-tagged ferritin-based nanoparticle (FNP) was biotinylated and then dialyzed to remove excess biotin. Subsequently, the biotinylated FNP was loaded onto streptavidin biosensors (ForteBio) until saturation. To measure the binding affinity of FNP to NiV G-Fc, the loaded streptavidin biosensors were dipped into 2-fold series of decreasing concentrations of NiV G-Fc proteins for 15min, followed by a 15min dissociation. Real-time data was analyzed using ForteBio Data Analysis 6.4 and kinetic curves and steady-state equilibrium were fitted using a global 1:1 binding algorithm with drifting baseline.

### Negative staining analysis

Negative-staining electron microscopy procedures were conducted as previously described. Briefly, the purified FNP sample (5 μL) with a final concentration of 0.15 mg/mL in PBS was loaded onto a freshly glow-discharged carbon-coated grid. After 1 min incubation, excess sample was blotted, and the grid was stained with 5 μL 2% (w/v) uranyl acetate solution for 1 min. Excess solution was blotted and grids were dried at room temperature. Images were acquired using an FEI Tecnai G2 20 TWIN electron microscope operated at 200 kV and at a magnification of 50,000×.

### Immunization of mice

Four-week-old C57BL/6J mice (Male, Vital River Laboratories) were immunized with FNP-NiV G protein (5 μg/mouse), NiV G protein (5 μg/mouse), or PBS buffer control in the presence of two adjuvants: aluminum hydroxide (Alum, 250μg/mouse; InvivoGen) and monophosphoryl lipid A (MPLA, 5 μg/mouse; InvivoGen) via intramuscular route (i.m.) in a volume of 50 μL. The immunized mice were boosted with the same dose of immunogen and adjuvants 3 weeks later. Sera from the immunized mice were collected on weeks 2, 4, 6 and 8 post the second immunization for the detection of specific IgG antibodies and analysis of neutralizing antibodies. Subsequently, sera were collected every month for up to 6 months after the second immunization for assessing long-term immunity.

### Enzyme-linked immunosorbent assay

Firstly, ELISA was conducted to assess the binding of Fc tagged NiV G (NiV G-Fc) protein to FNP, with ovalbumin (OVA, InvivoGen) used as negative control. ELISA plates were pre-coated overnight at 4°C with NiV G-Fc or OVA (2 μg/mL) and subsequently blocked with 5% skim milk in PBS for 1 h at 37°C. Following this, 8×His tagged FNP protein (0.5 μg/mL) was added to the wells and incubated for 2 h at 37°C. After four washes, the binding was detected using an HRP-labeled anti-His tag antibody (Beyotime) for 1 h at room temperature. The reaction was visualized by addition of substrate 3,3’,5,5’-Tetramethylbenzidine (TMB, Beyotime) and terminated with a stop solution without Sulfuric Acid (Beyotime). The absorbance at 450 nm (OD450) was measured using an ELISA plate reader (BioTek).

Subsequently, ELISA was carried out to investigate the binding of NiV G-Fc to soluble hEphrinB2 protein, and human IgG Fc (Fc, Sino Biological) protein used as a control. Briefly, ELISA plates were pre-coated with NiV G-Fc or Fc protein (2 μg/mL) overnight at 4°C and blocked with 5% skim milk in PBS for 1 h at 37°C. Serially diluted 6×His tagged hEphrinB2 protein was added to the plates and incubated for 2 h at 37°C. After four washes, the bound protein was detected using an HRP-labeled anti-His tag antibody for 1 h at 37°C. The reaction was visualized by addition of TMB and stop solution. The OD450 was measured by an ELISA plate reader.

Finally, ELISA was also performed to detect the interaction between NiV G protein and NiV G-specific antibodies in sera collected from mice and golden hamsters. The procedure mirrored that described above, with the modification that the ELISA plates were pre-coated with NiV G-His at 2 μg/mL. Subsequently, the plates were sequentially incubated with serially diluted mouse sera and HRP-conjugated anti-mouse antibodies (1:20000, Abcam). Plates were washed as before prior to being developed with TMB and stop solutions before reading OD450. The cutoff value was defined as 2.1-fold of OD450 values from the sample of nonvaccinated mice.

### Pseudovirus neutralization and inhibition assays

NiV and HeV pseudoviruses were generated, as previously described (51). Briefly, HEK293T cells were co-transfected with a plasmid encoding Env-defective, luciferase-expressing HIV-1 genome (pNL4–3.luc.R-E-) and plasmids encoding NiV or HeV G and F proteins corresponding to NiV-M and NiV-B isolates, utilizing the lipo2000 reagent (Thermo Fisher Scientific). The medium was replaced with fresh DMEM (supplemented with 2% FBS) 8 h post transfection. Pseudovirus-containing supernatants were collected 48 h later for a single-cycle infection in HEK293T cells. Subsequently, a pseudovirus neutralization assay was performed by incubating NiV or HeV pseudovirus with heat-inactivated (30 min at 56°C), 2-fold serially diluted mouse sera for 1 h at 37°C. The mixture was then added to HEK293T cells. After 48 h, the cells were lysed in cell lysis buffer (Promega), and the lysed cell supernatants were incubated with a luciferase substrate (Promega). Relative luciferase activity was detected, and the 50% pseudovirus neutralizing antibody titer (NT_50_) was calculated.

### Flow cytometry

Flow cytometry analysis was initially employed to assess the binding of the NiV G-Fc protein to hEphrinB2 expressing Raji cells with Fc protein was used as control. Briefly, cells were incubated with NiV G-Fc or Fc protein, which were serially diluted, for 30 min at room temperature. After three washes with PBS, the cells underwent incubation with goat anti-human IgG (H+L) conjugated with Alexa Fluor 488 (Invitrogen, A-11013) for 1h at 4°C. Subsequently, cells were washed three times and analyzed using flow cytometry (BD Biosciences). Binding efficiencies of NiV G-Fc were quantified as the percentage of Alexa Fluor 488-positive cells among mCherry-positive cells (EphrinB2-expressing cells).

Subsequent flow cytometry analysis aimed to detect the interaction between NiV G-Fc and hEphrinB2 in the presence of mouse sera. Briefly, hEphrinB2-Raji cells were incubated with NiV G-Fc (100 ng/mL) in the presence or absence of serially diluted mouse sera at room temperature for 1 h. This was followed by incubation with Alexa Fluor 488-conjugated goat anti-human IgG antibody (1:2000, Invitrogen) for 30 min and subsequent analysis.

### Enzyme-linked immunospot assay

To assess the responses of antigen-specific T lymphocytes, we conducted an IFN-γ-based ELISpot assay using a mouse IFN-γ ELISpot kit (Mabtech). Briefly, spleens were collected from C57BL/6J mice 2 weeks post the boost vaccination. Subsequently, splenocytes were isolated and stimulated with a pool of NiV G protein peptides (2 μg/mL of individual peptide) on 96-well plates pre-coated with mouse IFN-γ antibodies. Phorbol 12-myristate 13-acetate (PMA) and ionomycin (Dakewe) were induced as a positive control, while unstimulated cells served as the negative control. Following a 24 h of incubation, the plates were processed in turn with biotinylated IFN-γ-detection antibody, HRP conjugated streptavidin, and substrate according to the manufacturer’s protocols. The count of antigen-specific spots was subsequently determined using an automatic ELISPOT reader (AID GmbH).

### Live virus neutralization assay

The collected mouse sera were scrutinized for the presence of neutralizing antibodies against authentic viral infection by NiV-M (Malaysia strain) and NiV-B (Bangladesh strain) within biosafety level 4 (BSL4) facilities. Heat-inactivated mouse sera were serially diluted in 3-fold from 1:20, mixed with NiV-M or NiV-B (100 TCID_50_), and incubated at 37°C for 1 h. The mixtures were subsequently added to Vero cells pre-plated in 96-well tissue culture plates. Following incubation, the mixtures were added to Vero cells pre-plated in 96-well tissue culture plates in quadruplicate and cultured at 37 °C for five days. Cells with or without virus were used as positive or negative control, respectively. Cytopathic effect (CPE) of cells was recorded on day 5 post-infection. Neutralizing antibody titer (NT_50_) was expressed as the highest dilution of mouse sera capable of preventing virus-caused CPE in at least 50% of the wells in quadruplicate.

### Immunization and viral challenge with Syrian hamsters

Three groups of golden Syrian hamsters (6-week-old, Female, Vital River Laboratories) were immunized with either FNP-NiV G protein (10 μg, n=12), NiV G-Fc protein (10 μg, n=12), or PBS control (n=12). The immunization included the use of adjuvants identical to those employed in mice studies. Animals were primely vaccinated via intramuscular (i.m.) inoculation in a volume of 100 μL and boosted in a 3-week interval. Serum samples were collected at indicated time points for immunological analysis. Three weeks following the boost vaccination, all hamsters were transferred to animal biosafety level 4 (ABSL-4) facilities and challenged with 1000 LD_50_ of NiV-M strain in 500 μL DMEM via the intraperitoneal (i.p.) injection route. Six hamsters from each group were euthanized at 5 days post infection (dpi), and tissues from the brain, lung, and spleen were collected for viral load detection and histopathological analysis. The remaining six animals were monitored daily for changes in body weight, clinical signs of disease, and overall survival for a period of up to 21 days post challenge.

### RNA isolation and viral RNA load detection

Tissues for RNA isolation post dissection were weighed and subjected to homogenization. 140 μL of clarified tissue homogenate was added to 560 μL of AVL viral lysis buffer (Qiagen) for RNA extraction using the QIAamp Viral RNA Kit (Qiagen) following the manufacturer’s instructions. Extracted viral RNA was analyzed by qRT-PCR using HiScript II One Step qRT-PCR SYBR Green Kit (Vazyme) with NiV specific primers targeting NiV nucleocapsid (N) gene. The primes were as follows: NiV-N-Forward, 5’-CACAGAACTGCTCGGCACA-3’ and NiV-N-Reverse, 5’-ACATCAGCAGGAAGGCAAGAG -3’. Threshold cycle (Ct) values, indicative of viral genome loads, were analyzed using CFX Manager Software, and data were presented as viral RNA copies. To generate NiV RNA standards, an RNA transcript of the N gene, serving as the reference RNA, was transcribed *in vitro* from a linearized plasmid containing the NiV N gene. The copy number of reference RNA was calculated using Avogadro’s number and its molecular weight. The standard curve was constructed by plotting the Cq values against the known initial reference mRNA copy number. Quantitative calculations of RNA load per milligram of tissues were performed based on the respective tissue weights used for RNA extraction. RNA loads of samples that were undetected were defined as 100 copies, limit of detection (L.O.D) of developed qRT-PCR assay.

### Histopathology and immunohistochemistry analyses and scoring

Tissues of hamsters including brain, lung and spleen, were fixed in 10% formalin for one week, with two changes of fresh paraformaldehyde solution before transfer out of the ABSL-4 facility, following standard operating procedure approved by the Institutional Biosafety Committee. Subsequently, the samples were embedded in paraffin and sectioned to a thickness of 4µm. Hematoxylin and eosin (H&E) staining was employed for identifying histopathological changes in the brain, lung or spleen under light microscopy. Pathological evaluations were conducted blindly, employing a 4-point scoring system by a pathologist (27), with scores ranging from 0 (indicating no change) to 1–4 (indicating increasing severity).

For immunohistochemistry, NiV N protein was detected using a rabbit polyclonal antibody prepared in-house. Briefly, tissue sections were treated with anti-NiV-N primary antibody at a 1:1000 dilution at 4°C overnight. An HRP-labelled goat anti-rabbit IgG secondary antibody was applied at a 1:200 dilution for 50 min at room temperature, followed by a diaminobenzidine (DAB) chromogen reaction for approximately 15 s and counterstaining with hematoxylin for 45s. Image acquisition was performed using a Pannoramic MIDI system (3DHISTECH Ltd., HUN).

### Statistical analysis

The values are presented as mean ± standard error of the mean (SEM). Statistical analyses were conducted using GraphPad Prism 9.0 software. A Student’s two-tailed unpaired t test was performed to analyze the statistical differences between two experimental groups. For comparisons involving more than two experimental groups, one-way ANOVA with Dunn’s multiple comparisons was applied. Significance was set at P < 0.05, with “ns” indicating not significant. The following notation was used for levels of significance: *** P < 0.001, ** P < 0.01, * P < 0.05.

## Data availability statement

The original contributions presented in the study are included in the article/**Supplementary Materials**. Further inquiries can be directed to the corresponding author.

## Ethics statement

The animal study was approved by Institutional Animal Care and Use Committee of the Wuhan Institute of Virology, Chinese Academy of Sciences. The study was conducted in accordance with the local legislation and institutional requirements.

## Author contributions

CY: Writing – original draft, Visualization, Validation, Software, Resources, Project administration, Methodology, Investigation, Funding acquisition, Formal analysis, Data curation, Conceptualization. YY: Writing – review & editing, Resources, Methodology. PY: Writing – review & editing, Project administration. HL: Writing – review & editing, Project administration. GG: Writing – review & editing, Project administration. YP: Writing – review & editing, Project administration. MC: Writing – review & editing, Project administration. ML: Writing – review & editing, Project administration, Methodology. XZ: Writing – review & editing, Project administration. WG: Writing – review & editing, Project administration. ZZ: Writing – review & editing, Project administration. XH: Writing – review & editing, Resources. ZY: Writing – review & editing, Supervision, Funding acquisition. CS: Writing – review & editing, Supervision, Resources, Funding acquisition.

## Supplementary material

Supplementary Figure 1 Preparation of ferritin-based NiV G protein nanoparticle vaccine (FNP-NiV G) and subunit vaccine NiV G. Ferritin-based nanoparticles **(A)**, Fc-tagged NiV G of NiV-M **(B)**, and NiV-B **(C)**, and the FNP-NiV G complex **(D)** were purified using Superose 6 Increase 10/300 GL size exclusion chromatography (SEC), respectively. Elution profiles of the three proteins from Superose 6 Increase 10/300 GL size exclusion chromatography (SEC) are illustrated on the left, and representative SDS-PAGE gels stained with Coomassie blue of peak fractions from the SEC are listed on the right. Experiments were repeated twice with similar results.

Supplementary Figure 2 The NiV-M and NiV-B G-Fc proteins showed comparable and high potency in NiV receptor binding ability. **(A)** Detection of NiV-M-G-Fc and NiV-B-G-Fc bindings to hEphrinB2 by ELISA, respectively. The data are presented as the mean ± SEM (n=3). **(B)** Cellular surface binding of hEphrinB2 to Fc-tagged NiV-M and NiV-B G proteins by flow cytometry. Raji cells transduced with human EphrinB2 (hEphrinB2-Raji cells) were incubated with NiV G-Fc proteins for analysis of G binding activity, shown as the percentage of Alexa Fluor 488-stained cells. This experiment was independently repeated three times with similar results. Both Human IgG Fc protein and mock hEphrinB2-Raji cells acted as negative controls. **(C–D)** Binding affinity of NiV-M and NiV-B G proteins to human EphrinB2. Kinetic sensograms of NiV-M-G-Fc **(C)** and NiV-B-G-Fc **(D)** binding to human EphrinB2 fit a 1:1 binding model, as determined by BLI. EphrinB2 proteins were captured, and 2-fold serial dilutions of NiV G protein were then incubated. Experiments were performed three times with similar results, and one set of representative data is displayed.

Supplementary Figure 3 Analysis of neutralizing mechanisms induced by vaccine-generated antibodies. Representative images illustrate the receptor binding inhibition of NiV-M-G-Fc **(A)** and NiV-B-G-Fc **(B)** when exposed to sera (1:640) from mice immunized with PBS (left panel), NiV G (middle panel), or FNP-NiV G (right panel). The violet lines represent median fluorescence intensity (MFI) values. The dark red lines depict the binding interaction between NiV-M-G-Fc or NiV-B-G-Fc and hEphrinB2. The light gray shades indicate Fc-hEphrinB2 binding. All experiments were conducted in duplicate.

Supplementary Figure 4 Antibodies induced by the FNP-NiV G immunization in Syrian hamsters neutralize pseudotyped NiV variants. The cross-neutralizing antibodies from FNP-NiV G immunized sera (3-fold serial dilutions from 1:40) were assessed to interrupt the cellular entry of pseudoviruses of NiV-M **(A)** and NiV-B **(B)** in HEK293T cells. The data are presented as mean ± SEM (n=6). Statistical differences among the groups were analyzed using a Student’s two-tailed t-test.

Supplementary Figure 5 Pathological analysis of spleen tissue by HE staining. Foci of necrosis were present in the spleens of PBS group (black arrows), and the boundary between white pulp and red pulp became not clear, which was not observed in vaccine groups. Representative images for each group are shown at 20× magnification and scale bars indicate 500 μm.

Supplementary Table 1 Severity Scoring of the tissues (lung, spleen, brain) from hamsters post NiV challenge.

Supplementary Table 2 The information of Nipah virus isolates in this paper.
