# Supplementary figures and images for: A highly effective ferritin-based divalent nanoparticle vaccine shields Syrian hamsters against lethal Nipah virus
Source: Front Immunol. 2024 Jun 6;15:1387811. doi: 10.3389/fimmu.2024.1387811 (PMC11191641; doi:10.3389/fimmu.2024.1387811)

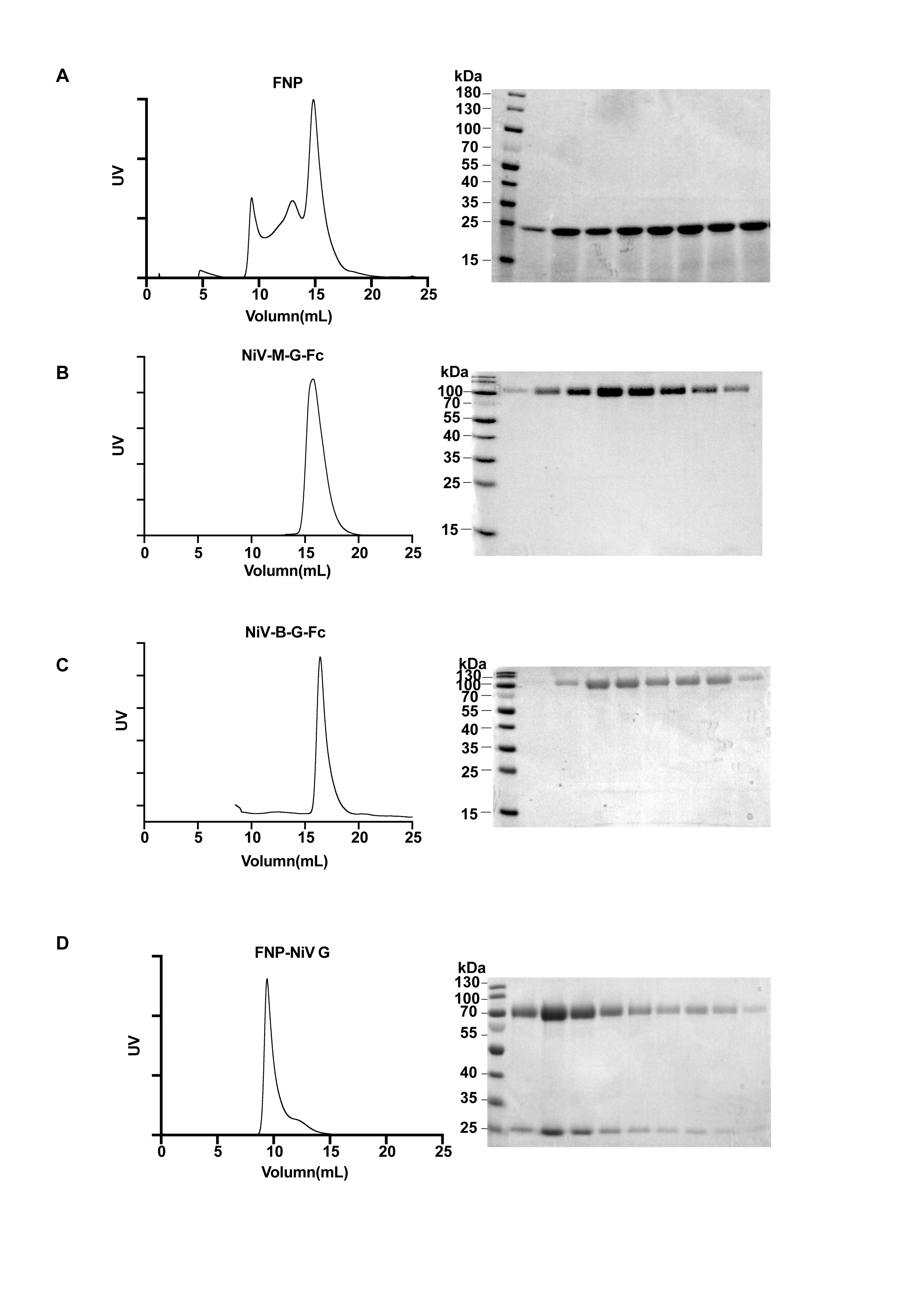

Supplement: Supplementary Figure 1 — Preparation of ferritin-based NiV G protein nanoparticle vaccine (FNP-NiV G) and subunit vaccine NiV G. Ferritin-based nanoparticles (A), Fc-tagged NiV G of NiV-M (B), and NiV-B (C), and the FNP-NiV G complex (D) were purified using Superose 6 Increase 10/300 GL size exclusion chromatography (SEC), respectively. Elution profiles of the three proteins from Superose 6 Increase 10/300 GL size exclusion chromatography (SEC) are illustrated on the left, and representative SDS-PAGE gels stained with Coomassie blue of peak fractions from the SEC are listed on the right. Experiments were repeated twice with similar results. [file Image_1.tif]

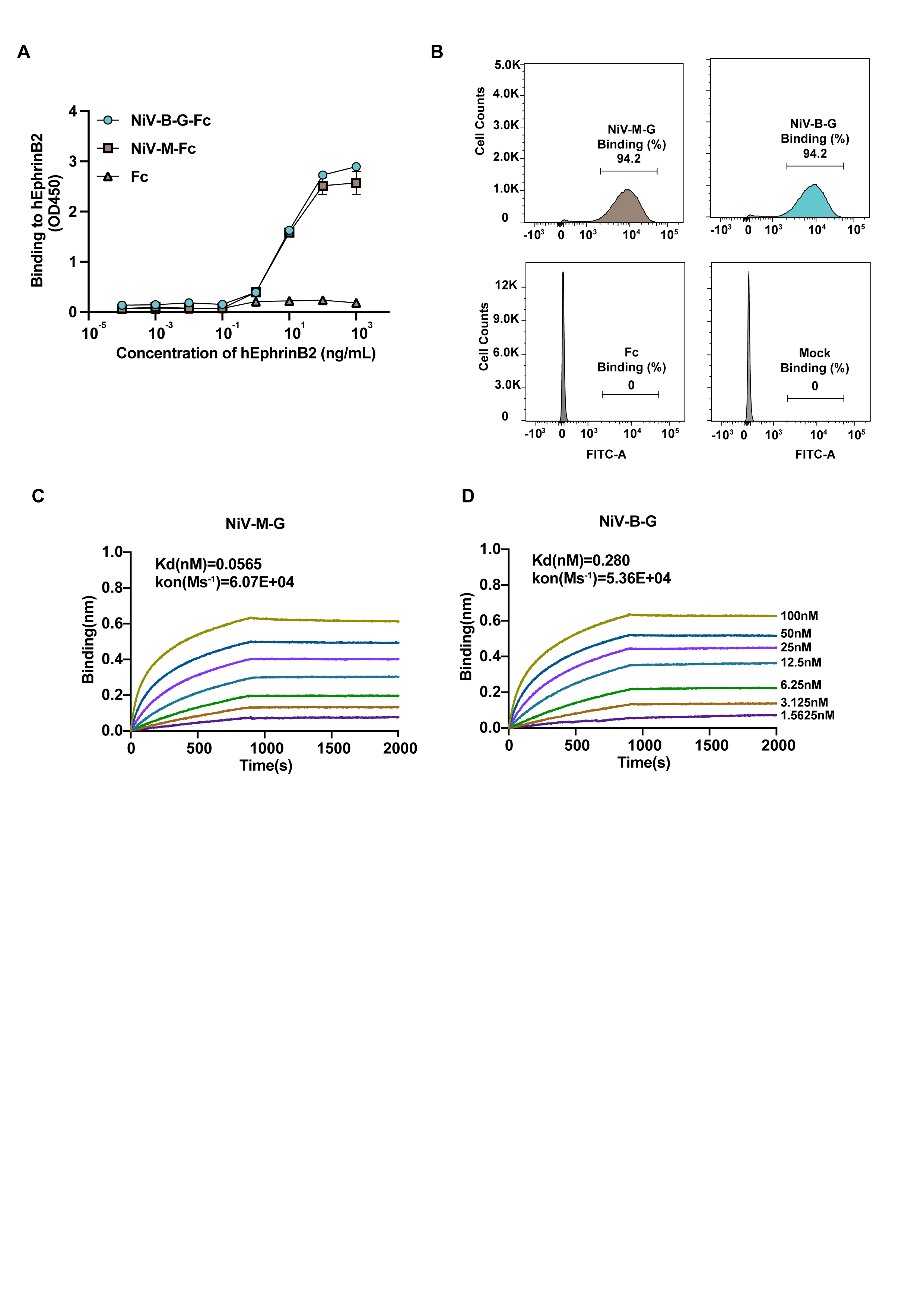

Supplement: Supplementary Figure 2 — The NiV-M and NiV-B G-Fc proteins showed comparable and high potency in NiV receptor binding ability. (A) Detection of NiV-M-G-Fc and NiV-B-G-Fc bindings to hEphrinB2 by ELISA, respectively. The data are presented as the mean ± SEM (n=3). (B) Cellular surface binding of hEphrinB2 to Fc-tagged NiV-M and NiV-B G proteins by flow cytometry. Raji cells transduced with human EphrinB2 (hEphrinB2-Raji cells) were incubated with NiV G-Fc proteins for analysis of G binding activity, shown as the percentage of Alexa Fluor 488-stained cells. This experiment was independently repeated three times with similar results. Both Human IgG Fc protein and mock hEphrinB2-Raji cells acted as negative controls. (C–D) Binding affinity of NiV-M and NiV-B G proteins to human EphrinB2. Kinetic sensograms of NiV-M-G-Fc (C) and NiV-B-G-Fc (D) binding to human EphrinB2 fit a 1:1 binding model, as determined by BLI. EphrinB2 proteins were captured, and 2-fold serial dilutions of NiV G protein were then incubated. Experiments were performed three times with similar results, and one set of representative data is displayed. [file Image_2.tif]

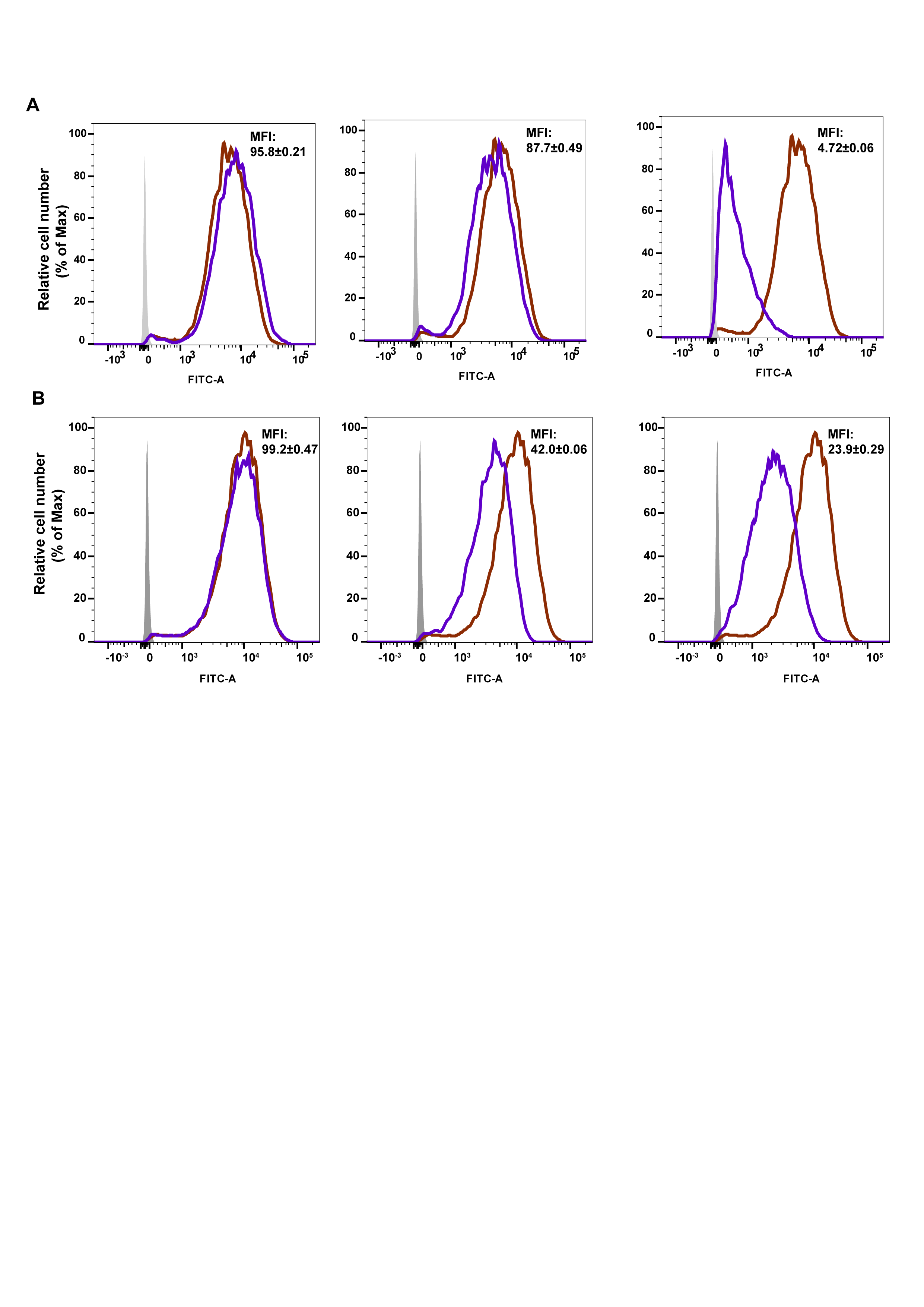

Supplement: Supplementary Figure 3 — Analysis of neutralizing mechanisms induced by vaccine-generated antibodies. Representative images illustrate the receptor binding inhibition of NiV-M-G-Fc (A) and NiV-B-G-Fc (B) when exposed to sera (1:640) from mice immunized with PBS (left panel), NiV G (middle panel), or FNP-NiV G (right panel). The violet lines represent median fluorescence intensity (MFI) values. The dark red lines depict the binding interaction between NiV-M-G-Fc or NiV-B-G-Fc and hEphrinB2. The light gray shades indicate Fc-hEphrinB2 binding. All experiments were conducted in duplicate. [file Image_3.tif]

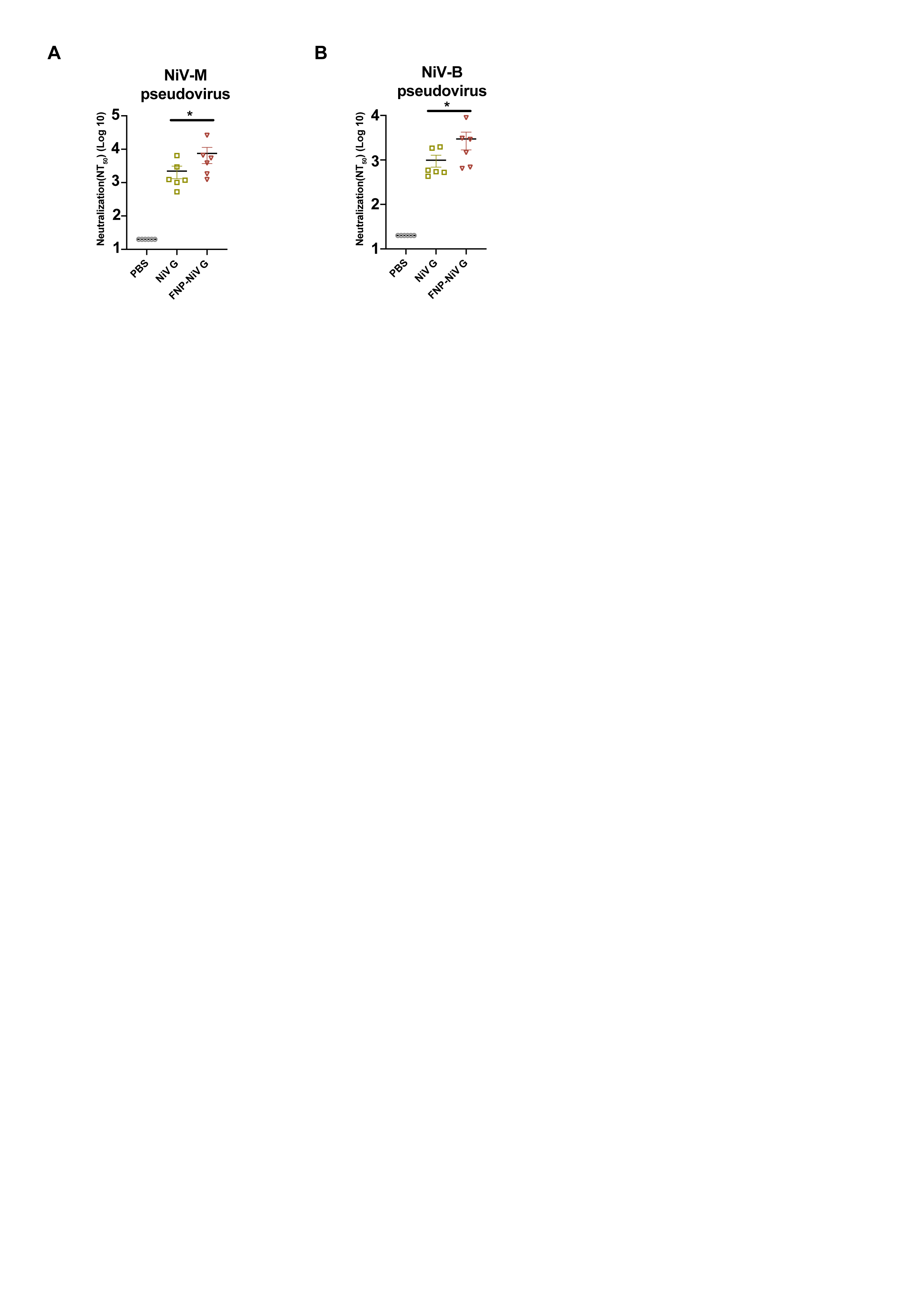

Supplement: Supplementary Figure 4 — Antibodies induced by the FNP-NiV G immunization in Syrian hamsters neutralize pseudotyped NiV variants. The cross-neutralizing antibodies from FNP-NiV G immunized sera (3-fold serial dilutions from 1:40) were assessed to interrupt the cellular entry of pseudoviruses of NiV-M (A) and NiV-B (B) in HEK293T cells. The data are presented as mean ± SEM (n=6). Statistical differences among the groups were analyzed using a Student’s two-tailed t-test. [file Image_4.tif]

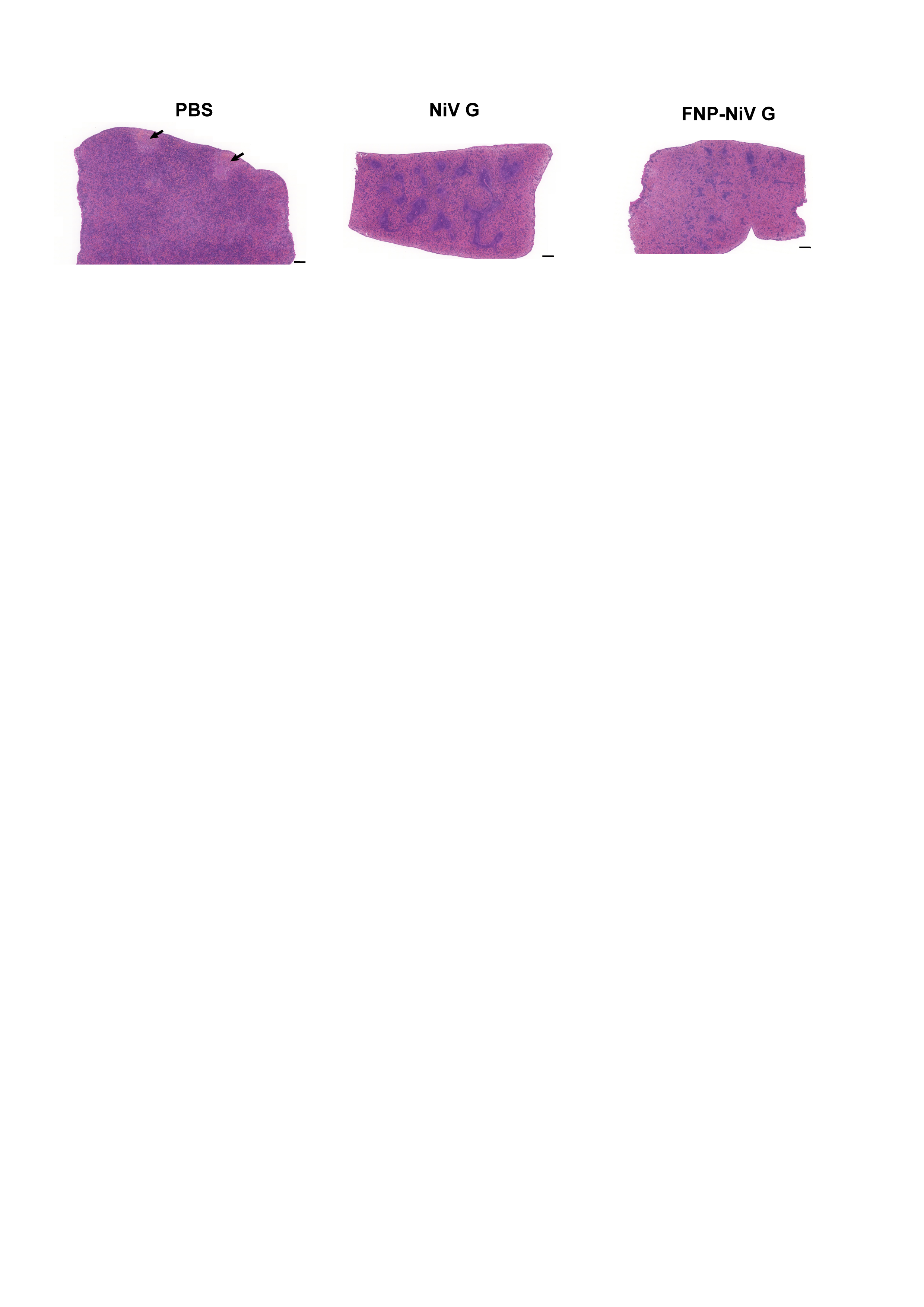

Supplement: Supplementary Figure 5 — Pathological analysis of spleen tissue by HE staining. Foci of necrosis were present in the spleens of PBS group (black arrows), and the boundary between white pulp and red pulp became not clear, which was not observed in vaccine groups. Representative images for each group are shown at 20× magnification and scale bars indicate 500 μm. [file Image_5.tif]
